# De Novo Esophageal Carcinoma in Post-liver Transplant Patient

**Published:** 2014-11-01

**Authors:** A. H. Tank, V. K. Sutariya, P. R. Modi

**Affiliations:** Department of Gastrointestinal Surgery and Liver Transplantation, Institute of Kidney Diseases and Research Centre, Institute of Transplantation Science, Civil Hospital, Ahemdabad, Gujarat, India

**Keywords:** Carcinoma, squamous cell, Esophageal neoplasms, Humans, Liver diseases, alcoholic, Liver Transplantation, Neoplasms, Esophageal squamous cell carcinoma, Tobacco use

## Abstract

*De novo* esophageal malignancy following liver transplantation is very rare. Esophageal squamous cell carcinoma following liver transplant is closely associated with history of alcohol intake and tobacco chewing. We report on a 45-year-old man, chronic tobacco chewer and alcoholic who underwent liver transplantation for alcoholic cirrhosis and developed esophageal squamous cell carcinoma 23 months following the procedure. He was treated surgically and has had a tumor-free survival after 34 months of regular follow-up.

## CASE REPORT

A 45-year-old man who was tobacco chewer, smoker and alcoholic presented with progressive dysphagia for solids since two months before. He underwent liver transplantation for alcoholic cirrhosis 23 months before. The patient had stopped alcohol consumption but continued tobacco chewing following the transplantation. He was given steroid as induction therapy during liver transplantation that gradually shifted to two-drug oral therapy at presentation—tacrolimus 1 g/day and prednisolone 5 mg/day. He developed post-transplantation new-onset diabetes mellitus.

**Figure 1:**
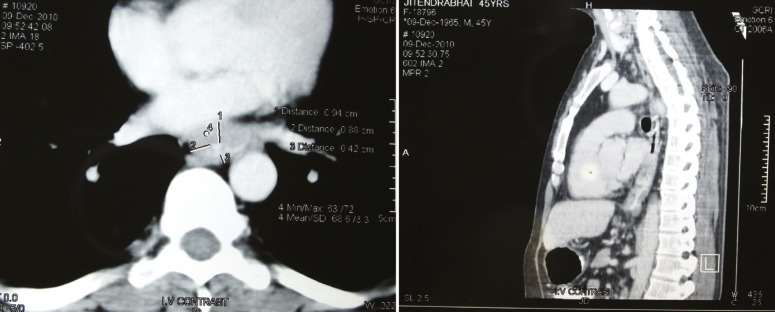
Computed tomography of the patient suggesting esophageal wall thickening without any lymphadenopathy

At esophagoscopy, he had an ulcerated mass involving two walls at 26–32 cm from dental arcade causing partial obstruction of the lumen; scope passed beyond the lesion where no other abnormality was detected. Biopsy of the lesion shows well differentiated squamous cell carcinoma. Computed tomography (CT) revealed esophagus wall thickening of 15.8 mm, involving approximately 5 cm segment in the lower esophagus; no lymphadenopathy or other lesions were found (Fig 1). Contrast study showed an irregular filling defect at the middle and lower third of the esophagus; the rest of the esophagus and stomach appeared normal.

Considering no local or distant metastasis, the patient was fit to undergo surgery. A midline laparotomy done and thoracolaproscopic-assisted mobilization of esophagus was done; then, esophagectomy, two-field lymhadenectomy and cervical esophagogatric anastomosis were done and feeding jejunostomy was inserted.

Per-op finding was suggestive of growth of 5 cm at mid-esophagus involving whole esophageal circumference. Pathological examination revealed a moderately differentiated squamous cell carcinoma involving 5.5 cm in length, whole circumference, and full thickness of esophagus deep to adventitia; all 13 lymph nodes resected were free from tumor.

The patient had an uneventful early post-op period. However, he developed stricture at the anastomosis site requiring dilatation for five times at 15-day intervals, after which he was able to take diet orally. He did not receive any adjuvant therapy. Patient developed hernia from the midline incision after one year that was treated by meshplasty. At present, 34 months post-operative, he has no tumor or any associated symptoms.

## DISCUSSION

Liver transplantation is a life-saving procedure for cirrhotic patients, but requires life-long immunosuppression which carries many side effects including increased risk of malignancy. Risk of malignancy following other solid organ transplantation such as kidney is well documented, but it is less well understood post-liver transplantation. Some studies are available showing risk of *de novo* malignancy after liver transplantation is two-fold higher than general population, ranging from 4%– 16% [1, 3].

Esophageal squamous cell carcinoma is uncommon malignancy following liver transplantation and few cases have so far been reported. Squamous cell carcinoma of esophagus is closely associated with history of alcoholism and tobacco chewing, however, the risk would increase after immunosuppression due to its carcinogenic effect [4]. The mechanisms through which long-term immunosuppression would increase the risk of malignancy is well-known. However, if the patient has extra risk factors such as alcohol and tobacco consumption, the chance of development of early and unusual malignancies like squamous cell carcinoma would be increased [5].

Our patient had undergone pre-op esophagoscopy, which did not show any malignancy. In post-transplantation period, he continued tobacco chewing and 23 months following the transplantation he found to have squamous cell carcinoma of the esophagus. There are reports of cases with esophageal carcinoma that developed soon after they received liver transplants for alcoholic cirrhosis [5, 6]. Increased incidence of squamous cell carcinoma in oropharengeal cavity is also documented in patients who underwent liver transplantation for alcoholic cirrhosis [4]. Rapid development of high grade dysplasia or adenocarcinoma in Barrett’s esophagus is also documented in post-liver transplant patients [7]. Definite increased risk of malignancy is documented in such group of patient but time of development is not well defined.

Our patient was treated with surgery alone without any adjuvant therapy; the tumor was completely resected; no recurrence occurred after almost 36 months. The patient had no distant metastasis at the time of presentation, which was favourable. Furthermore, on histopathological examination, no lymph nodes were involved, so early diagnosis is the only key to cure of esophageal carcinoma. Sabine, *et al*, showed that those who require adjuvant therapy for distant metastasis at the time of presentation had less favourable outcome than those who treated surgically [6]. Therefore, all patients who underwent liver transplantation for alcoholic cirrhosis should be carefully screened for early diagnosis of esophageal malignancies.
